# Age-associated alterations in CXCL1 chemokine expression by murine B cells

**DOI:** 10.1186/1471-2172-5-15

**Published:** 2004-07-26

**Authors:** Lina Hu, Vishwa Deep Dixit, Valeria de Mello-Coelho, Dennis D Taub

**Affiliations:** 1 Laboratory of Immunology, Gerontology Research Center, National Institute on Aging-Intramural Research Program, National Institutes of Health, 5600 Nathan Shock Drive, Baltimore, MD 21224, USA

**Keywords:** Chemokines, Aging, Lymphocytes, B cells, immunodeficiency, CXCL1

## Abstract

**Background:**

The CXCL1 chemokines, macrophage inflammatory protein-2 (MIP-2) and cytokine-induced neutrophil chemoattractant (KC), have been shown to play a role in a number of pathophysiological disease states including endotoxin-induced inflammation and bacterial meningitis. While the expression of these chemokines has been identified in a variety of cell types in the mouse, little is known about their expression with murine B-lymphocytes.

**Results:**

Here, we demonstrate that highly purified murine splenic B cells are capable of expressing both MIP-2 and KC protein and mRNA upon activation with lipopolysaccharide (LPS) but not in response to anti-μ and anti-CD40 in combination with interleukin-4 (IL-4) stimulation. Moreover, these chemokines are expressed at higher levels in B cells derived from young (4 m) compared to old (24–29 m) mice. Upon fractionation into distinct B-cell subsets, we found that the expression of MIP-2 and KC by aged follicular (FO) B cells is significantly decreased when compared to the same cells from younger mice, while only MIP-2 production was found to be diminished in aged marginal zone (MZ) B cells. Interestingly, MIP-2 and KC production by newly formed (NF) B cells did not significantly differ with age. Moreover, the potential relevance of these findings is supported by the poor ability of LPS-activated aged B cells to specifically mediate CXCL1-dependent leukocyte recruitment when compared to younger B cells.

**Conclusion:**

Overall, the decreased expression of CXCL1 chemokines by aged B cells in response to LPS may have potential implications on the secondary recruitment of leukocytes to sites of microbial infections and inflammation possibly contributing to the increased susceptibility of older subjects to pathogen challenge.

## Background

Chemokines are a superfamily of small chemotactic proteins that have been classified into four major subfamilies, namely CXC, CC, C, and CX3C, based on the presence or absence and positional arrangement of N-terminal cysteine (C) residues [1-3]. One of the hallmarks of chemokine function is to facilitate trafficking and recirculation of immune cells from the circulation and tissues into secondary lymphatic organs and various peripheral tissues to maintain immune homeostasis *in vivo *[4]. These ligands also control the selective recruitment of specific leukocyte subsets to sites of inflammation and immune reactions. Besides migration, chemokines also induce the rapid activation of integrin molecules. The two CXC chemokines, macrophage inflammatory protein-2 (MIP-2) and cytokine-induced neutrophil chemoattractant (KC), are members of the CXCL1 subfamily containing a glutamate-leucine-arginine (ELR) motif that are well known for their ability to induce the activation and recruitment of neutrophils *in vitro *and *in vivo *[5-7]. These chemokines are also believed to be the murine structural and functional homologues of human CXCL8, IL-8 and chemokine growth-related oncogene (GRO) [8,9]. MIP-2 and KC display high affinity binding and signaling through the murine CXCR2, a 7-transmembrane G protein-coupled receptor [10].

It has been well established that endotoxin and various proinflammatory cytokines (e.g., TNF-α and IL-1) stimulate the expression of MIP-2 by macrophages, neutrophils and epithelial cells [11-13]. Numerous studies have also demonstrated a pathophysiological role for MIP-2 and KC in several inflammatory disease states including endotoxemia-induced lung injury [14], 1996), glomerulonephritis [15], bacterial meningitis [16] and herpes simplex virus type 1 (HSV-1) infection [17]. In mice, the cellular sources of MIP-2 have been confirmed to be macrophages [6], epithelial cells [12], bone marrow endothelial cells [18], astrocytes [19] and mast cells [20]. In humans, in addition to macrophages, monocytes, T, NK and B cells have also been shown to produce and respond to CXCL8 [21-24]. Despite all of these reports, few studies have focused on chemokine production by B cells and the relevance of such production in cell-mediated immune responses.

Age-related dysfunction of the immune system has often been attributed to a variety of measurable changes in the functional activity of many immunomodulatory factors. This immune deterioration with age is believed to contribute to the morbidity and mortality in humans, possibly due to the greater incidence of infection, autoimmunity and cancer in the elderly. Dysregulation of lymphocyte function is thought to play a critical part in these processes. Many factors are believed to contribute to age-associated immunodeficiencies including defects in cellular signaling, stem cell and bone marrow defects, thymus involution, alterations in hormone and growth factor production, and replicative senescence. Chemokines are believed to play a pivotal role in the complex communication network between different cell types that enable the selective trafficking of many immune effector cells to the necessary sites at the appropriate times. Several reports have also demonstrated increased expression of inflammatory cytokines and chemokines in the circulation and by peripheral blood cells with age suggesting that uncontrolled inflammation may contribute to the increased susceptibility to infection and injury in certain aged cohorts. For example, IL-8 chemokine production by human T, NK and monocytes is altered with age in response to bacteria products [25-27]. Alterations in chemokine production and responsiveness may have a significant impact on the capacity of aged subjects to control or even mount an immune response.

The objective of the current study is to explore both young and aged murine splenic B cells and B cell subsets for their ability to express the CXC chemokines, MIP-2 and KC, in response to LPS and other stimuli. Three B cell subpopulations were studied here including: (1) Marginal zone (MZ) B cells, which are uniquely positioned in the marginal sinus where they interact with efficient Ag trapping circulating cells and are thought to be involved in the early response against thymus-independent (TI) blood-borne Ags [28,29]; (2) Follicular (FO) B cells, which are long-lived recruiting B cells located at the border of the B cells follicle and the T cell containing PALS zone that facilitate responses to thymus-dependent (TD) Ags and give rise to both germinal center (GC) B cells and plasma cells [30]; and (3) Newly formed (NF) B cells, which correspond to the recently immigrated B cells from the bone marrow and have been described as immature/transitional B cells and the precursors of FO and MZ B cells [31]. We also investigated the effect of these B cell-derived chemokines on splenocyte migration and the influence of aging on this event. The relevance of these findings to the generation of humoral and cellular immune responses shall be discussed.

## Results and Discussion

**Figure 1:**
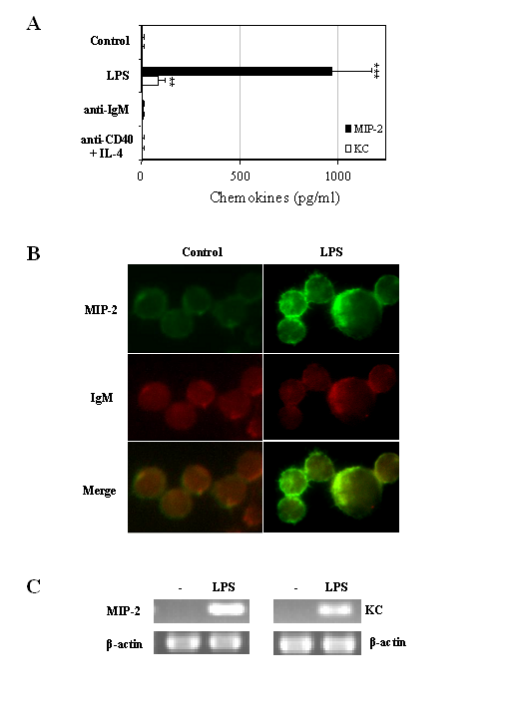
**Splenic B cells produce MIP-2 and KC in response to LPS. **(A) Purified splenic B cells (1 × 10^6 ^cells/ml) from 4 month-old C57BL/6 mice were stimulated with 10 μg/ml of LPS, anti-IgM or anti-CD40 plus IL-4 for 24 h. Then, cell-free supernatants were collected and assayed by ELISA for MIP-2 and KC secretion. Data are mean of triplicate ± SD of one representative of two experiments. The value was significantly different from non-stimulated control. (**, *P *< 0.01; ***, *p *< 0.005) (B) Immunofluorescence visualization of MIP-2 expression in the cytoplasm of 24 h LPS-stimulated splenic B cells. Cells were incubated with biotinylated goat anti-mouse MIP-2 antibody followed by SA-Oregon green-488 (green), PE-conjugated anti-IgM antibody (red) and the DNA dye DAPI (blue not shown) and subjected to cytospin (5 × 10^5 ^cells/microscope slide). Magnification: +100. (C) Total RNA was isolated from non- and LPS-stimulated cells and levels of MIP-2 and KC mRNA were examined by RT-PCR. The housekeeping gene β-actin was amplified as an internal control. The data are representative of two experiments.

As expression of MIP-2 and KC by murine splenic B cells has not been previously defined, we initially examined whether murine B cells are capable of producing the CXCL1 chemokines, MIP-2 and KC, upon culture or post cellular activation with LPS, anti-IgM or CD40 mAb in combination with IL-4 for 24 h. Culture supernatants were subsequently examined for MIP-2 and KC expression using ELISA analysis. The results shown in Figure 1A demonstrate that while cultured non-stimulated splenic B cells failed to spontaneously produce detectable levels of MIP-2 and KC (<10 pg/ml), stimulation of B cells with LPS, but not anti-IgM or CD40 mAb in combination with IL-4, resulted in the significant increase in the expressions of both of these chemokines. Interestingly, the quantity of MIP-2 produced in response to LPS was significantly greater than KC. Fig. 1B shows representative images of MIP-2 expression patterns in the cytoplasm of LPS-stimulated splenic B cells. Visually, accumulation of cytoplasmic MIP-2 was observed in splenic B cells (IgM^+^) following 24 h incubation with LPS, but was less evident in non-stimulated splenic B cells. Similar results were also obtained examining cytoplasmic KC (data not shown). In agreement with the above findings, MIP-2 and KC mRNA was only detected in LPS- (Fig. 1C) but not anti-μ- or anti-CD40 mAb/IL-4-stimulated (data not shown) B cells. Consistent with protein expression, the mRNA levels for MIP-2 were expressed at a greater level than KC. As the engagement of the B cell receptor (BCR) initiates signaling pathways mediated through nonreceptor protein tyrosine kinases, including Fyn, Lyn, Syk, and Bruton's tyrosine kinase (BTK), while LPS activate B cells by stimulating signaling through toll-like receptors, more specifically TLR4. The CC chemokines, MIP-1α and MIP-1β, have been shown to be induced and secreted by human B cells in response to BCR signaling [22]. In contrast, the lack of CXCL1 chemokine induction in response to cross-linking of the BCR with soluble anti-IgM antibody here suggests that the BCR signal(s) may not be optimal and/or essential for MIP-2 and KC production. Perhaps additional costimulatory signals or activation pathways may be necessary for CXCL1 production in combination with BCR signaling. These data demonstrate that murine splenic B cells are capable of secreting the CXCL1 ligands upon activation with LPS but not via anti-μ or anti-CD40 stimulation.

**Figure 2:**
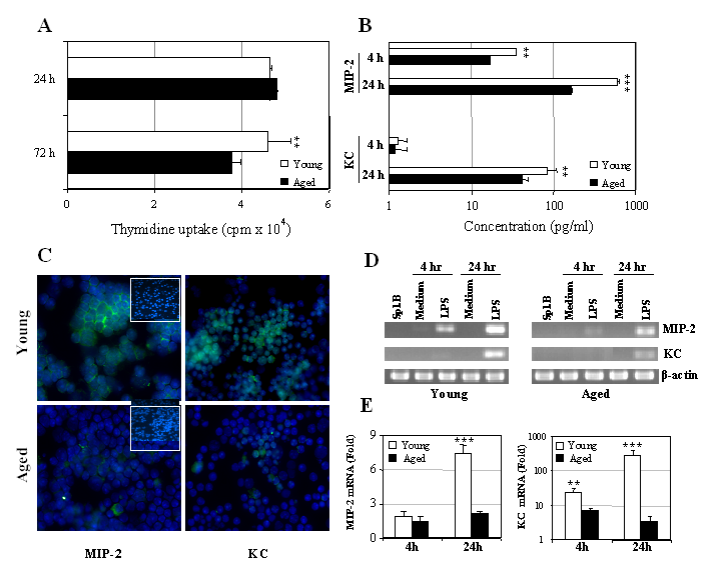
**Effect of aging on LPS-induced B cell proliferation and MIP-2 and KC production by splenic B cells. **(A) *In vitro *proliferation of splenic B cells stimulated with LPS. Purified splenic B cells (1.25 × 10^5^/ml) were cultured with or without LPS. Proliferation was measured by [^3^H] thymidine uptake after 24 and 72 h of culture. Data are means ± SD of three mice in each group. The value was significantly different from that of aged mice. (** P < 0.01) (B) Splenic B cells from three to five young and aged mice were stimulated with 10 μg/ml of LPS for 4 and 24 h. After stimulation, cell-free supernatants were collected and assayed by ELISA for MIP-2 and KC secretion. One representative experiment out of three is shown. (C) 24 h LPS-stimulated splenic B cells were subjected to immunofluorescence staining with anti-MIP-2 and anti-KC antibodies and DNA dye DAPI as described in Figure 1. One representative experiment out of three is shown. Control anti-goat IgG staining in both young and aged B cells are shown as inserts in this panel. (D) After stimulation, cells were harvested, and RNA was prepared for MIP-2 and KC specific RT-PCR. The housekeeping gene β-actin was amplified as an internal control. Data shown are representative of two independent experiments. (E) MIP-2 and KC mRNA levels were measured by real time RT-PCR and normalized to threshold cycle (*Ct*) values of the co-amplified house-keeping gene GAPDH. Normalized values were calibrated to the value derived from non-stimulated controls and shown as fold change of mRNA expression. Data shown are representative of two independent experiments. Value were significantly different from those in aged mice (** P < 0.01; *** P < 0.005)

Several reports have demonstrated that the proliferative response of whole spleen cells to LPS declines with age [32,33]. The results in Figure 2A demonstrate that purified splenic B cells derived from aged mice also display a significantly diminished proliferative response when cultured with LPS for 72 h when compared to B cells derived from younger mice. However, no significant differences in cellular proliferation were observed between young and aged mice after 24 h of culture, which is in accordance with the previous report [34].

Despite no significant differences in LPS proliferative responses between young an aged mice at 24 h, LPS-stimulated B cells derived from young mice demonstrated approximately 4 times greater levels of MIP-2 in the culture supernatants than aged B cells during this same time period. Similarly, reduced levels of KC expression were also detected in the 24 h culture supernatants LPS-stimulated B cells derived from aged mice when compared to younger animals (Fig. 2B). Moreover, as shown in Fig. 2C, splenic B cells from aged mice demonstrate significantly less expression of intracytoplasmic MIP-2 and KC upon stimulation with LPS when compared to younger B cells. These results were supported by conventional RT-PCR analysis where MIP-2 and KC mRNA signals were barely detectable in both freshly isolated and non-stimulated splenic B cells from young or aged mice. The mRNA levels of MIP-2 and KC chemokines in LPS-stimulated B cells from aged mice were dramatically lower (> 2-fold) than those in younger mice (Fig. 2D). Unlike MIP-2, the KC mRNA signal was only detected after 24 h of stimulation with LPS and demonstrated similar patterns of age-associated expression as MIP-2. Furthermore, real time RT-PCR analysis also demonstrated similar age-related alterations in B cell-derived MIP-2 and KC mRNA expression, albeit after 24 h of LPS stimulation (Fig. 2E).

**Figure 3:**
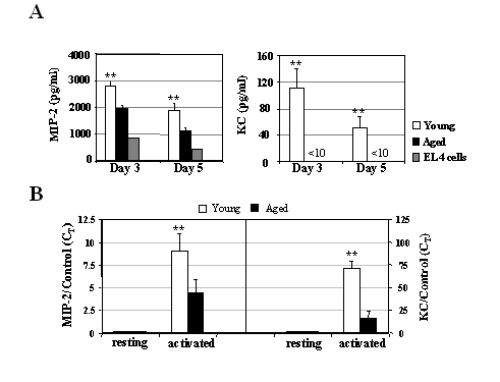
**Effect of aging on MIP-2 and KC production by splenic B cells co-cultured with irradiated EL-4 cells. **(A) Decreased amounts of MIP-2 and KC proteins in supernatants of splenic B cells cultured in EL-4 culture system. Splenic B cells (3 × 10^4^/well) from three to five young and aged mice were cultured with irradiated EL-4 thymoma cells in the presence of LPS and macrophage supernatant for 3 or 5 days. Culture supernatants were collected and analyzed by ELISA for MIP-2 and KC secretion. These values were significantly different from those in aged mice (** P < 0.01). (B) Real-time RT-PCR analysis of MIP-2 and KC mRNA expression in activated splenic B cells from young and aged mice. After 3 days of culture, total RNA was isolated from cultured cells and levels of MIP-2 and KC mRNA were measured by real time RT-PCR and normalized to threshold cycle (*Ct*) values of the co-amplified housekeeping gene GAPDH. Normalized values were calibrated to the value derived from EL-4 only controls and expressed as fold induction of mRNA. One representative experiment out of two is shown.

Similar results were obtained using an *in vitro *B cell culture system in which murine naïve B cells can be activated and induced to proliferate and differentiate into Ab-forming cells. The EL-4 system has been described as a potent *in vitro *culture system for B activation, proliferation and differentiation [35-37]. Naive B cells cultured in the EL-4 system differentiate to Ig-secreting cells expressing switched isotypes and a plasma cell phenotype (Fig. 5; [38,39]). The presence of both LPS-dependent and T cell-dependent stimulation (e.g., CD40-CD40L) contribute to the plasma cell differentiation in this model system. Here, splenic B cells derived from young and aged mice were co-cultured with irradiated EL-4 thymoma cells in presence of LPS and a macrophage-derived culture supernatant. MIP-2 and KC levels were assessed in the supernatants of those cultures via ELISA on days 3 or 5. As shown in Fig. 3A, splenic B cells cultured in this EL-4 culture system yielded similar results to those observed in Fig. 2. Briefly, a considerable reduction of MIP-2 production by aged splenic B cells was observed in comparison to B cells derived from younger animals. Interestingly, no KC expression was detected in any of the aged B cell cultures while significant KC production was observed in the 3 and 5-day culture supernatant of young B cells. Furthermore, real time RT-PCR also demonstrated a significant reduction in the mRNA expression of MIP-2 and KC in LPS-activated aged B cells when compared to younger B cell cultures (Fig. 3B). Moreover, given our data in Figure 1 demonstrating that cross-linking of CD40 by anti-CD40 antibody and IL-4 failed to elicit CXCL1 chemokine secretion by B cells, the B cell-derived MIP-2/KC secretion in EL-4 system may be attributed to the effect of LPS-dependent signals rather than CD40 cross-linking signals and/or other cytokines/lymphokines presented in the culture. Taken together, these results demonstrate that MIP-2 and KC expression by murine splenic B cells is significantly altered with increasing age.

**Figure 4:**
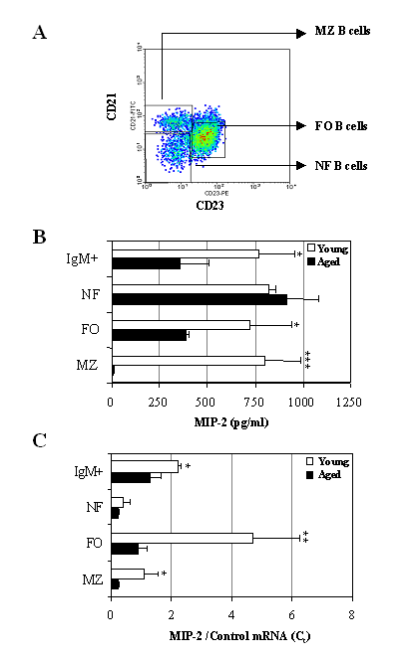
**Ability of distinct splenic B cell subpopulations from young and aged mice to produce MIP-2 and KC chemokines. **(A) Spleen cells were isolated from three young and aged mice, and then stained with anti-IgM, anti-CD23 and anti-CD21 Abs. Subsequently, NF, FO and MZ B cell subpopulations were sorted from IgM^+ ^gated cell population. (B) Amounts of MIP-2 protein in supernatants of total IgM^+ ^B cells and distinct splenic B cell subpopulations cultured in EL-4 system. NF, FO and MZ B cells within the respective gates shown were directly sorted into individual wells of 96 well plates (1,000 cells/well). Each well had 200 μl of medium containing irradiated EL-4 cells, LPS and macrophage supernatant. After 5 days of culture, supernatants were collected and analyzed by ELISA for MIP-2 and KC secretion. These values were significantly different from those in aged mice (* P < 0.05; ** P < 0.01; *** P < 0.005). (C) Real time RT-PCR analysis of MIP-2 and KC mRNA expression in 5 days-cultured B cells from young and aged mice. MIP-2 and KC mRNA levels in total IgM^+^, NF, FO and MZ B cells were measured by real time RT-PCR and normalized to threshold cycle (*C*_*t*_) values of the co-amplified housekeeping gene GAPDH. Normalized values were calibrated to the value derived from EL-4 only controls and expressed as fold induction of mRNA. One representative experiment out of two is shown. These values were significantly different from those in aged mice (* P < 0.05; ** P < 0.01; *** P < 0.005).

Splenic B cells derived from adult mice are comprised of several distinctive subpopulations based on their surface marker expression [40]. To assess if the CXCL1 ligand expression differences observed between young and aged mice are due to differences in B cell subsets, MZ, FO, and NF B cells as well as total IgM^+ ^B cells were sorted by FACS based on their surface IgM, CD21, and CD23 markers (Fig. 4A). Sorted individual B cell subpopulations were co-cultured with EL-4 cells for 5 days. Similar to our above results, total IgM^+ ^splenic B cells derived from aged mice expressed significantly reduced levels of MIP-2 compared to young B cells. Moreover, although MIP-2 secretion by NF B cells derived from aged mice did not significantly differ from young NF B cells, the levels of MIP-2 expressed by aged FO B cells were substantially diminished in comparison to B cells derived from their younger counterparts. Furthermore, unlike MZ B cells derived from young mice, MIP-2 production was almost undetectable in MZ B cells obtained from aged mice (Fig. 4B). In support of these data, MIP-2 mRNA expression was significantly reduced in aged FO and MZ B cells but not in aged NF B cells when compared to B cells derived from younger mice (Fig. 4C). Similar results were observed on day 3 (data not shown). Unfortunately, in several repeated subset studies, we were unable to detect KC in the culture supernatants of B cell subsets derived from young and aged mice. We believe this may be due to the low level of KC produced by the splenic B cell subsets compared to non-sorted, non-fractionated splenic B cells, which were also quite low albeit detectable. B cells stimulated with EL-4 T cells behave differently than LPS-stimulated primary B cells. B cells co-cultured with EL-4 cells demonstrate better viability and more plasmocytic differentiation than LPS alone. Thus, B cells are in different states of activation in these two culture systems and may account for the differences in KC expression.

**Figure 5:**
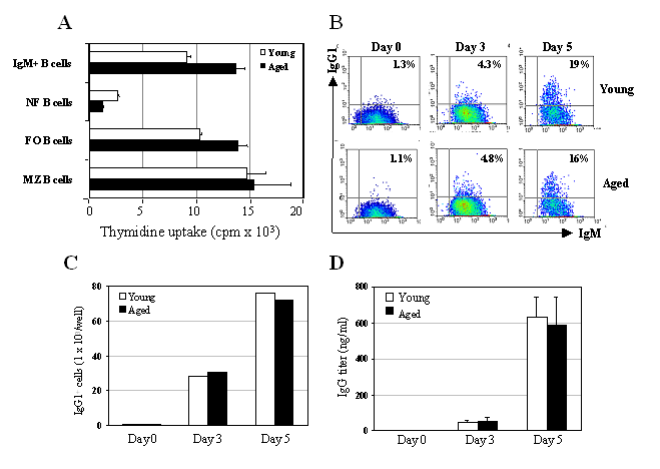
**Similar proliferation and differentiation between young and aged splenic B cells cultured in EL-4. **FACS-sorted distinct B cell subsets (1000/well) from three young and aged mice were cultured in EL-4 culture system. Proliferation was measured by [3H] thymidine uptake after 3 days culture. Data represent the mean and variations (SD) from triplicate cultures. The data presented are representative of two independent experiments (A). Purified splenic B cells (4 × 10^4^/well) from three young and aged mice were cultured with irradiated EL-4 thymoma cells in the presence of LPS and macrophage supernatant for 3 and 5 days. The cultured cells were stained with anti-IgG1 and anti IgM Abs. Percentages of IgG1^+ ^IgM^+ ^B cells are indicated (B). Absolute numbers of IgG1^+ ^B cells in culture. Total numbers of IgG1^+ ^B cells per well were calculated from a mixture of 20 wells in each group (C). Culture supernatants were measured by ELISA for IgG production. Data are representative of two independent experiments (D).

Given the possibility that the reduced MIP-2 and KC expression by aged B cells in these EL-4 cultures might reflect a diminished capacity of these cells to proliferate and/or differentiate, we examined the proliferative response as well as the generation of class switched B cells in the culture of those B cells. By culturing MZ, FO and NF B cell subpopulations as well as IgM^+ ^B cells on EL-4, no defect in the cell proliferations was found among those distinct B cell subsets in aged mice on day 3 (Fig. 5A) and day 5 (data not shown) of culture. It should be noted that the EL-4 cells utilized in these assays were thymidine kinase deficient [41] and thus, the background thymidine incorporation was quite low (<200 cpm).

On the other hand, analysis of both percentages (Fig. 5B) and the absolute number of IgG1^+^ B cells (Fig. 5C) revealed that the frequency of IgG1^+ ^B cells presented in the aged B cell culture were comparable to those generated in the young B cell culture in response to LPS. Furthermore, ELISA analysis of Ig levels in culture supernatants demonstrated no difference in the level of IgG produced by young and aged splenic B cells in response to LPS (Fig. 5D). These studies suggest that the reduced MIP-2 and KC production observed in EL-4 B cell subset cultures are not due to significant alterations in cellular proliferation and/or differentiation.

**Figure 6:**
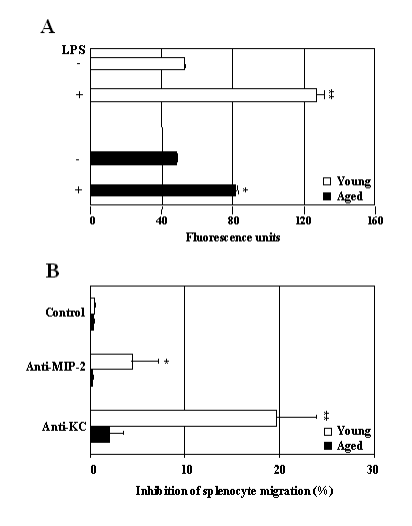
**B cell-derived MIP-2 and KC induce splenocyte migration. **(A). Purified splenic B cells (1.5 × 10^6^/ml) from three young and aged mice were added to the lower chamber of Transwell plates and stimulated with and without LPS. 24 hr after LPS stimulation, spleen cells from 4 month old C57BL/6 mice were preincubated with Hoechst and subjected to chemotaxis through 5-μm pore size Transwell filters (upper chamber) to the supernatants in the lower chambers. Hoechst fluorescence of accumulated cells in the lower chamber was measured. Data are the mean of triplicate cultures ± SD of one representative of two experiments. The value was significantly different from that of control. (* P < 0.05; ** P < 0.01) (B). For the neutralization, Abs against MIP-2 and KC were added to the supernatants in the lower chambers at the beginning of culture. The percent inhibition was calculated as follows: 100 - 100 × (chemotaxis with neutralizing Ab/chemotaxis without neutralizing Ab). Data are the mean of triplicate cultures ± SD of one representative of two experiments. The value was significantly different from that of control (* P < 0.05; ** P < 0.01).

To assess the potential functional relevance of MIP-2 and KC expression by LPS-stimulated splenic B cells, splenocytes derived from young mice were labeled with Hoechst and examined for their ability to migrate in response to culture supernatants derived from LPS-stimulated young and aged cells. The results in Fig. 6A show that culture supernatants of LPS-stimulated splenic B cells from either young and aged mice induced significant responder cell migration, as compared with non stimulated B cells-derived supernatants. More importantly, the chemotactic activity of culture supernatants derived from LPS-activated younger B cells was significantly higher than the activity observed in response to supernatants derived from LPS-stimulated aged B cells. Interestingly, the addition of anti-MIP-2 Ab to the B cell cultures resulted in an approximate 5% reduction of the migratory capacity of the young responder cells to migrate in response to the LPS-derived supernatants of young B cells but this antibody addition failed to exhibit any significant inhibition of migration induced by aged B cell supernatants (Fig. 6B). Neutralization of responder cell migration was also significantly blocked with anti-KC Ab (~20%) in the young but not aged B cell supernatants. Additional neutralization studies using a panel of anti-chemokine antibodies revealed that several CC chemokines are also being made by B cells and are playing a role in the remaining chemotaxis observed using these young responder cell populations (data not shown). Overall, these results suggest that B cell-derived MIP-2 and KC may play a possible role in leukocyte trafficking and that the MIP-2 and KC derived from these B cell cultures are biologically active.

In the current study, we report that endotoxin-activated murine B cells express and secrete the CXC chemokines, MIP-2 and KC. These results are in accordance with the previous studies, which demonstrate that activated human B cells express and secrete CXCL8 [21-23]. We also demonstrate that the expression of CXCL1 chemokines, MIP-2 and KC, decline with age in murine splenocytes and B cells, particularly evident in MZ and FO B cell subsets (Fig. 2, 3 and 4). This could not be attributed to impaired numbers of MZ and FO B cells as judged by flow cytometric analysis as young and aged B cells demonstrated comparable numbers of these populations (data not shown). In addition, these differences could also not be attributed to the alterations in proliferation (Fig. 2A and 5A; [34]) or terminal differentiation of these cells to IgG plasma cells (Fig. 5B,5C and 5D). Thus, the downregulation of MIP-2/KC chemokine production in response to LPS by MZ and FO B cells from aged in relation to young mice appears to be due to an age-dependent signaling difference in response to LPS. In this respect, the expression and function of the LPS receptor TLR4 has been shown impaired in aged animals [42]. In addition, aging can affect gene regulation and some transcription factors, such as nuclear factor-kappa B (NF-kappa B) are required for induction by LPS of MIP-2/KC expression through TLR4 [43]. Thus, further studies will be necessary to elucidate the relevance of age-related alterations in TLR4 expression and/or function as well as NFκB-dependent transcriptional control in the age-related decline of CXCL1 expression in murine B cells.

The accumulation of MIP-2 and KC in tissues is known as an important event in early host defense against bacteria infection. Moreover, evidence indicates the MZ B cells are involved in the early stages of immune response against TI type 2 (TI-2) Ags derived from a number of encapsulated bacteria, including *Streptococcus pneumoniae*, *Neisseria meningitides*, and *Haemophilus influenzae *[44,45]. MZ B cells generate an early IgM producing plasma response after *in vivo *stimulation with TI antigen [29]. The remarkable correlation between the ability of MZ B cells to mount an immune response against bacterial-associated antigens and our observation showing that these cells produce neutrophil-attracting chemokines MIP-2/KC in response to LPS indicate a potential dual role for MZ B cells in preventing host from bacterial infection. Of particular significance for the functionality of MZ B cells, we found that MZ B cell-derived MIP-2 and KC expression was impaired in aged mice. Although TI-2-specific Ab immune responses were not found to be significantly altered with age [46], the diminished capacity of aging immune system to mount an optimal antibody response to encapsulated microbes could be attributed, at least in part, to the diminished capacity of lymphocytes to express inflammatory cytokines and chemokines, such as MIP-2 and KC. Alterations in CXCL1 chemokine production by aged B cells may also have implications in the secondary recruitment of granulocytes to local immune responses at peripheral sites or even within secondary lymphatic organs such as the mucosal immune system in the aged host.

Murine MIP-2 and KC exhibit similar expression patterns and functional activities to that of human IL-8 in inflammatory response. For example, it has been previously shown that a significant increase of IL-8 or MIP-2 in human and mice, respectively, occurs in the grain dust-induced inflammation of the lower respiratory tract [47]. Although age-related alterations in IL-8 production by human B cells have not yet been described, diminished expression of IL-8 has been observed in cultured IL-2-stimulated NK cells [25] and LPS-stimulated monocytes [27] derived from elderly subjects. These data suggest that an age-related MIP-2 or IL-8 production could be consequence of a defective functional activity of B cells in aging.

It should also be noticed that human IL-8 has been shown to be a potent chemoattractant for human B cells [23,24]. In the present study, we demonstrate that chemotactic activity of culture supernatant from young B cells was dramatically higher than that from aged B cells (Fig. 6A). Neutralization of these chemokines effects with addition of anti-MIP-2 and anti-KC Abs to the cultures resulted in a significant reduction of the migratory capacity of spleen cells to the culture supernatant from young, but not from aged B cells (Fig. 6B). This may be due to the low levels of MIP-2 and KC present in the culture supernatants of aged B cells that were not sufficient to induce significant migration of spleen cells to them. In addition, antibodies specific to KC and MIP-2 partially blocked the young splenic B cell-derived chemotactic activity toward splenocytes for approximately 20% and 5%, respectively, although high level of MIP-2 was present in the culture supernatants. The low ability of neutralizing anti-MIP-2 Ab to alter splenocyte migration suggests that MIP-2 produced by B cells may be a weaker migratory factor for murine splenocytes in relation to KC. As MZ B cells in spleen produce both MIP-2 and KC, one could hypothesize that these chemokines may facilitate B cell migration into and within the MZ area and/or amplify their activity and thus contributing to host defense.

## Conclusion

In summary, we demonstrate for the first time that murine splenic B cells are highly efficient in producing ELR-positive CXC inflammatory chemokines, in particular MIP-2, upon activation by LPS stimulation. Moreover, MIP-2 production, particularly by MZ B cells, was found to decline with age. Our finding suggests a possible linkage between functional activity of MZ B cells in production of neutrophil-attracting inflammatory chemokines and host defense. However, detailed and well-controlled *in vivo *studies will be necessary to assess these various possibilities and the significance of this CXCL1 production defect by aged B cells.

## Methods

### Mice

Specific pathogen-free 3–5 months (young) and 24–29 months (aged) C57BL/6 mice were purchased through the Office of Biological Resources and Resource Development of the National Institute on Aging (Bethesda, MD). All mice were maintained in an AAALAC-certified barrier facility and were acclimated for 2 weeks prior to use. All mice were fed autoclaved food and water *ad libitum*. All mice with evidence of disease (e.g., enlarged spleen, gross tumors) were not utilized in these studies.

### Preparation of splenic B cells

Splenic B cells were negatively selected via depletion of non-B lineage cells from spleen cells using a MACS system. Briefly, spleen cells were incubated with magnetic microbeads coated with anti-CD43 antibody and anti-CD11b antibody (Miltenyi Biotec, Bergisch Gladbach, Germany) at 4°C for 15 min, after which the cells were passed over a MACS apparatus. The purity of splenic B cells was consistently >95% as routinely checked by FACS analysis.

### ELISA analysis

Supernatants were collected from cultures and were then frozen at -80°C. The frozen supernatants were thawed at room temperature and chemokine levels were measured with commercial ELISA assay kits for MIP-2 and KC (R and D Systems, Minneapolis, MN) and immunoglobulins (Igs) (Bethyl, Montgomery, TX) according to the manufacturers' instructions.

### Immunofluorescence staining

Purified splenic B cells derived from young and aged C57BL/6 mice were cultured in the presence of absence of LPS (10 μg/ml) for 24 h at 37°C in 5% CO_2_. After culture, the cells were harvested, washed, fixed and permeabilized using 3.7% paraformaldehyde and 0.1% Triton X-100 for 15 min. After thoroughly washing these cells, non-specific binding sites were blocked using a 2% BSA solution containing 1% goat, rabbit serum and normal mouse IgG for 15 min at room temperature. Post incubation, these cells were incubated overnight at 4°C in presence of biotinylated mouse anti-MIP-2 and -KC antibodies (1 μg/ml) (R & D Biosystems, Minneapolis, MN). Streptavidin-conjugated Oregon green-488 (Molecular Probes, Eugene, OR) was utilized to label these cells the following day at a concentration of 1:250 for a period of 45 min at room temperature. After washing, cells were then labeled with PE-conjugated anti-IgM antibody (331,12; PharMingen) for 30 min., followed by the stains of cellular nuclei with DAPI (Molecular Probes, Eugene, OR) at concentration of 1 μg/ml for 10 min. These cells were subsequently placed into cytospin funnels and spun onto glass slides using a cytospin centrifuge (Shandon, Pittsburgh, PA) at 1200 rpm for 5 minutes. After being mounted in Immuno Fluor medium (ICN Biomedicals, Aurora, OH), images were acquired by Spot Advanced software on a Zeiss Axiovert S100 microscope under 100X objective (Carl Zeiss, Thornwood, NY).

### RT-PCR analysis

For conventional RT-PCR analysis, total RNA was extracted from cells using the RNeasy Mini kit (Qiagen, Valencia, CA) and cDNA was prepared from 1 μg of total RNA transcribed by the SuperScript First-strand Synthesis system for the RT-PCR procedure (Invitrogen, Carlsbad, CA) according to the manufacture's instructions. The mouse MIP-2 and KC primers (Sigma Genosys, Woodlands, TX) utilized in these studies were: MIP-2 sense 5'-TGCCGGCTCCTCAGTGCTG-3' and MIP-2 antisense 5'-AAACTTTTTGACCGCCCTTGA-3'; KC sense 5'-CGCTCGCTTCTCTGTGCA-3'and KC antisense 5'-ATTTTCTGAACCAAGGGAGCT-3' as described previously [48]. The cycling conditions for PCR were 95°C for 4 min for denaturation, followed by 30 cycles at 95°C for 30 s, annealing at 57°C for 45 s plus extension at 72°C for 45 s and a final 10 min at 72°C. After 30 cycles of the PCR, 10 μl of the PCR products were separated on a 1.8% agarose gel, stained with ethidium bromide, and photographs were taken. Their densities were quantified by using the image-analysis system FluorChem (Alpha Innotech Corporation, San Leandro, CA) and normalized using β-Actin housekeeping gene in the same sample.

### Real-time PCR analysis

Approximately 1 μg of total RNA was reverse transcribed by using SuperScript First-strand Synthesis system (Invitrogen, Carlsbad, CA) as described above. Real-time primers for murine MIP-2 and KC were designed using Primer Express software (Applied Biosystems) using the sequences from GenBank (MIP-2, accession no. X53798; KC, accession no. J04596; and GAPDH, accession no. NM_008084). Primers were constructed as follows: MIP-2 (forward primer, AGTGAACTGCGCTGTCAATGC; reverse primer, AGGCAAACTTTTTGACCGCC), KC (forward primer, TGCACCCAAACCGAAGTCAT; reverse primer, TTGTCAGAAGCCAGCGTTCAC), and GAPDH (forward primer, TGCATGGCCGTTCTTAGTTG; reverse primer, AGTTAGCATGCCAGAGTCTCGTT). Reverse-transcribed cDNA was amplified with primer sets for murine MIP-2, KC and GAPDH as indicated above using SYBR Green PCR core reagents and the GeneAmp 5700 Sequence Detection System (PE Applied Biosystems) following the manufacturer's instructions. No PCR products were generated from genomic versus cDNA template. Fold induction of mRNA was determined from the threshold cycle (*Ct*) values normalized for GAPDH expression and then normalized to the value derived from controls.

### EL-4-based B cell culture

The splenic B cell culture was performed using the EL-4-based B cell culture system as previously described [38]. Briefly, individual wells of 96-well flat-bottom plates were loaded with 5 × 10^4 ^irradiated (5,000 cGy) murine EL-4 thymoma cells (clone B5) in 200 μl of RPMI 1640 medium supplemented with 10% FCS, 10^-5^ M 2-mercaptoethanol (2-ME), 25 mM HEPES buffer, penicillin (100 U/ml), streptomycin (100 μg/ml), 10 μg/ml LPS, and 10% supernatant from culture of J774A.1 macrophage cells (no. TIB-67; American Type Culture Collection, Manassas, VA). MACS purified splenic B cells or FACS sorted B cells were seeded directly onto a feeder layer of irradiated EL-4 cells and cultured at 37°C in 5% CO2. Moreover, we have previously shown that approximately 97% of wells contained sorted single cells using the same outfitted FACStar Plus. These results were verified based on the resulting sequencing histograms demonstrating evidence of only one V6 light chain sequence in the amplified cDNA [38]. Therefore, any differences in the levels of CXCL1 chemokine secretion among B cell subsets and age should not be a consequence of unequal number of cells sorted in the culture plates. The culture supernatants were collected on day 3 or day 5 and tested for chemokine secretion by ELISA. These time points were selected based on optimal cell viabilities and time to permit cellular activation and proliferation.

### Flow cytometric analysis and cell sorting

The monoclonal Abs utilized for the cell surface staining were FITC-anti-CD21 (clone 7G6; PharMingen, San Diego, CA), -anti-IgM (331,12; PharMingen), PE-anti-CD23 (PharMingen), APC-anti-IgM (clone II/41; PharMingen), and/or biotinylated anti-IgG1 (PharMingen). Biotin-labeled antibody binding was visualized using UltraAvidin-R-Phycoerythrin (Linco Technologies, St. Louis, MO). For each staining, 10^6^ cells were pre-incubated with the blocking antibodies, the anti-Fc receptor (24G2), for 30 minutes on ice and then incubated with a mixture of mAbs for an additional 15 min on ice. Post washing, the cells were subsequently incubated with PE-streptavidin for 15 min on ice after which the cells were washed with 5% FCS/HBSS. These stained cells were subsequently analyzed on a Becton Dickinson FACScan flow cytometer using CellQuest software.

The B cell subsets, NF, FO and MZ, were isolated through the use of cell sorting using the combination of anti-IgM-APC, anti-CD23-FITC and anti-CD21-PE mAbs. Single spleen cell suspensions were stained with the aforementioned Abs and the MZ, FO and NF B cells within the gate of IgM^+ ^cell population were sorted based on their differential expression of CD21 and CD23 using a FACStar Plus (Becton Dickinson, San Jose, CA). The purity of each sorted population was consistently >95%.

### Proliferation assay

MACS-purified splenic B cells (1.25 × 10^5^/well) were cultured with 10 μg/ml of LPS for 1 and 3 days or FACS-sorted distinctive B cell subpopulations (1000/well) were cultured in EL-4 culture system for 3 and 5 days at 37°C in a 5% CO2. Cultures were pulsed with 1 μCi [^3^H] thymidine (NEN, Boston, MA) for the final 18 h. Cells were harvested on fiberglass paper. [^3^H] thymidine uptake was measured in a liquid scintillation counter (Beckman, Fullerton, CA).

### Chemotaxis assay

In the lower chambers of Transwell plates (Costar, Cambridge, MA), 1.5 × 10^6^/ml purified splenic B cells derived from young and aged mice suspended in 600 μl of RPMI 1640 medium supplemented with 2% FCS, 10-^5^ M 2-mercaptoethanol (2-ME), penicillin (100 U/ml), streptomycin (100 μg/ml) were cultured in the presence and in the absence of LPS (10 μg/ml) and incubated at 37°C in 5% in CO_2 _for 24 hr. After incubation, the cells and supernatants in the lower chambers were assessed for chemotactic activity using young splenocyte responder cells. These splenocytes were isolated from 4 month old C57Bl/6 mice after which they were preincubated with 10 μM Hoechst 33342 (Molecular Probes, Eugene, OR) in RPMI 1640 supplemented with 10% FCS for 30 min at 37°C. Subsequently, the Hoechst-stained splenocytes (1 × 10^6^) were washed with RPMI 1640 supplemented with 2% FCS, resuspended in 100 mL of medium, and then added to the upper chamber, containing a 6.5-mm diameter polycarbonate Transwell culture insert with 5 μm size pore. Each expected group was performed in duplicates with supernatants in the lower chambers for 8 to 10 h at 37°C. In certain assays, neutralizing antibodies specific for MIP-2 and KC at (1 μg/ml) were added to the low chamber containing the LPS-stimulated B cell supernatants. The transmigration of the Hoechst-labeled cells into the lower chamber were measured in a fluorescent spectrophotometer at 350 nm (excitation)/460 nm (emission). The results are expressed as fluorescent units or as the percentage inhibition of Hoechst-labeled splenocyte migration.

### Statistical analysis

Statistical evaluation of significance between the experimental groups was determined by Student's t test.

## List of abbreviations

Ag, antigen; MIP-2, Macrophage inflammatory protein-2; ELR, glutamate-leucine-arginine; GRO, growth-related oncogene; HSV-1, herpes simplex virus type 1; KC, cytokine-induced neutrophil chemoattractant; MZ, marginal zone; FO, follicular; NF, newly formed; TI, Thymus-independent; TD, Thymus-dependent

## Authors' contributions

LH, VMC, and VDD performed the experiments. LH and DDT prepared the figures and wrote the paper. DDT also supervised the work and edited the manuscript. All authors have read and approved the final manuscript.
